# Inflammasome-independent role of NLRP12 in suppressing colonic inflammation regulated by Blimp-1

**DOI:** 10.18632/oncotarget.8872

**Published:** 2016-04-20

**Authors:** Fushan Shi, Yang Yang, Mohammed Kouadir, Wei Xu, Songhua Hu, Tiancheng Wang

**Affiliations:** ^1^ Zhejiang Provincial Key Laboratory of Preventive Veterinary Medicine, College of Animal Sciences, Zhejiang University, Hangzhou 310058, China; ^2^ College of Animal Science and Technology, Zhejiang A&F University, Lin'an 311300, China; ^3^ Trustchem Co., Ltd., Nanjing 210029, China; ^4^ Department of Veterinary Medicine, College of Animal Sciences, Zhejiang University, Hangzhou 310058, China

**Keywords:** colitis, inflammation, Blimp-1, NLRP12, TLR4

## Abstract

NLRP12 is a member of the Nod-like receptor (NLR). Previous studies have reported enhanced colitis-associated inflammatory responses in NLRP12-deficient mice. In this study, we sought to investigate the role of NLRP12 in DSS-stimulated proinflammatory response in dendritic cells and mice colitis, and the molecular mechanisms involved in the development of the inflammation. Our results showed that down-regulation of NLRP12 is required for DSS-induced release of proinflammatory cytokines IL-1β and TNF-α; that PR domain zinc finger protein 1 (also known as Blimp-1) induces NLRP12 down-regulation during DSS-induced proinflammatory response and colitis; and that TLR4 is implicated in the up-regulation of Blimp-1 that led to the down-regulation of NLRP12 expression in DSS-induced colitis. Taken together, the results suggest that the TLR4-Blimp-1 axis promotes DSS induced experimental colitis through the down-regulation of NLRP12.

## INTRODUCTION

Ulcerative colitis is a disease that is characterized by inflammation in the lining of the large intestine. It usually affects the lower section of the intestine and the rectum, is associated with increased expression of several pro-inflammatory cytokines, including IL-1β, TNF-α and IL-6 [1, 2], and increases the risk of the development of colorectal cancer [3–5]. Experimental colitis in mice induced by dextran sodium sulfate (DSS) is similar to human ulcerative colitis, and is characterized by weight loss, diarrhea, bloody faeces, and tissue inflammation [6].

NACHT, LRR and PYD domains-containing protein 12 (NLRP12) is a member of the Nod-like receptor family (NLR), which has been largely characterized as activators of inflammation. As a member of NLR family, NLRP12 interacts with ASC (apoptosis-associated speck-like protein containing a CARD) [7] to form the NLRP12 inflammasome that promotes caspase-1 activation, which leads to the release of mature IL-1β. However, the physiological role of NLRP12 in the development of inflammation is still controversial, as both inflammatory and anti-inflammatory functions have been attributed to NLRP12. Recently, several studies have investigated the role of NRLP12 on colonic inflammation. It has been reported that NLRP12 can dampen inflammatory responses after colitis induction and suppress colitis-associated tumorigenesis, and that induction of colitis in NLRP12-deficient mice can lead to enhanced cytokine and chemokine release and promote hyperplasia and tumorigenesis [8]. However, the upstream regulator of NLRP12 during the development of colitis has still not been identified.

B lymphocyte-induced maturation protein-1 (Blimp-1), a zinc finger-containing transcriptional repressor, also known as PRDM1 (PR domain-containing 1, with ZNF domain), is a DNA binding factor which induces promoter silencing by recruiting histone deacetylases, histone arginine methyltranserses, histone lysine methyltransferases and co-repressors [9–11]. It is expressed in several cell lineages including B [12–14] and T cells [15, 16], macrophages [17], dendritic cells [18, 19], epithelial cells [20], and retinal neurons [21], and regulates cell differentiation through repression of several transcription activators. Blimp-1 was also shown to participate in the development of colitis; its deficiency in dendritic cells induces increased production of IL-6 and IL-1β upon MDP stimulation, and this phenotype is obvious in colonic DCs but not in BMDCs [22].

In this study, we sought to identify the upstream regulator of NLRP12 involvement in the development of DSS-induced colitis, and found that TLR4-mediated up-regulation of Blimp-1 led to the down-regulation of NLRP12 expression in DSS-induced colitis.

## RESULTS

### DSS treatment leads to a partially caspase-1 dependent release of IL-1β in murine DCs

**Figure 1:**
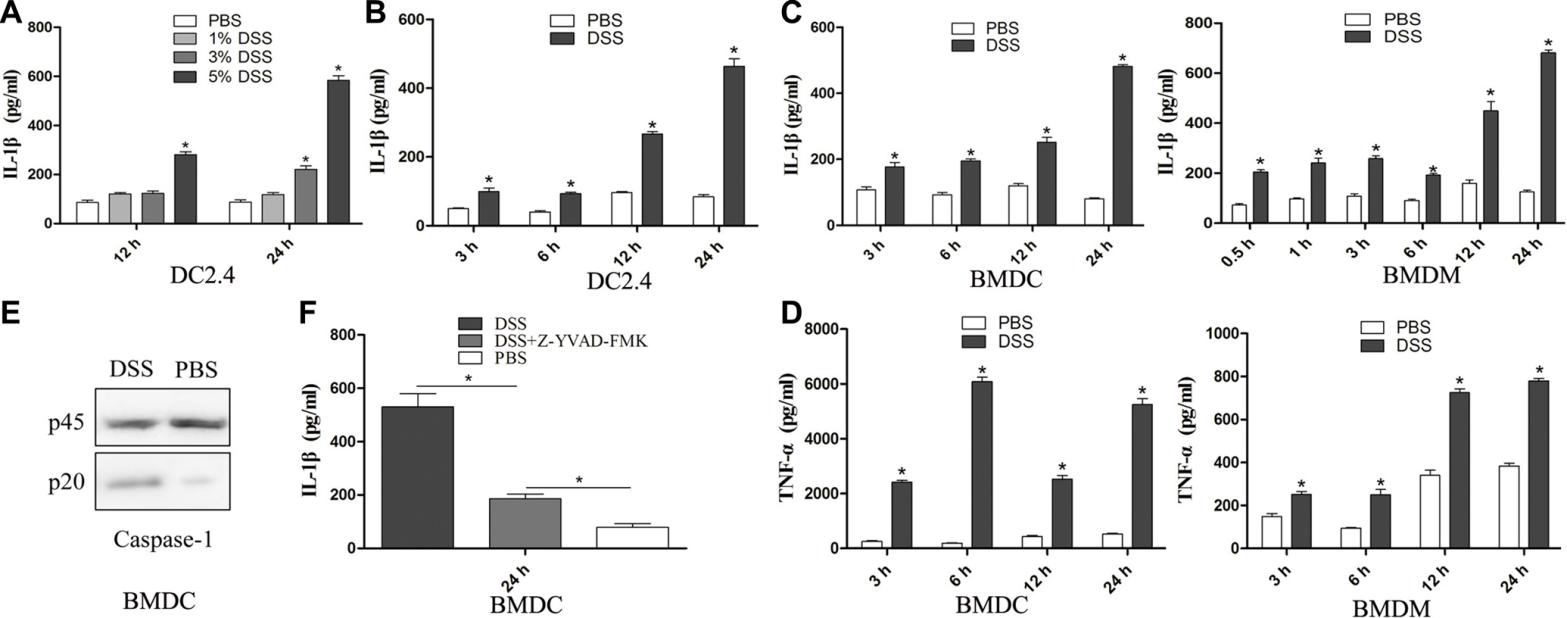
DSS induces partially caspase-1 dependent release of IL-1β in murine dendritic cells (**A**) DC2.4 cell lines were stimulated with different concentrations of DSS for 12 h or 24 h. IL-1β concentration in the supernatant was determined by ELISA. (**B**) DC2.4 cell lines were treated with 5% DSS at the indicated time points, and IL-1β was determined in the supernatant by ELISA. Detection of IL-1β (**C**) and TNF-α (**D**) release in BMDCs and BMDMs upon 5% DSS treatment at different time points. (**E**) Western blot analysis of pro-caspase-1 p45 and bioactive form of caspase-1 p20 in BMDCs after DSS stimulation. (**F**) Effect of the caspase-1 specific inhibitor Z-YVAD-FMK on IL-1β release in response to DSS treatment in mice BMDCs. Data represent the mean ± SD of triplicate samples from one of three independent experiments. *p < 0.05, significantly different from control under the same experimental conditions.

In order to investigate the mechanism of IL-1β release, we first incubated a murine dendritic cell line DC2.4 with different concentrations of DSS (1%, 3% and 5%, w/v) for 12 h and 24 h. As shown in Figure 1A, 5% of DSS treatment induced a significant release of IL-1β at the different time points examined (Figure 1B). Next, we stimulated murine primary BMDDC and BMDM cells with 5% DSS at different time points, and the results showed that 5% DSS stimulation significantly induced IL-1β release both from BMDCs and BMDMs at the indicated time points (Figure 1C). Furthermore, DSS also induced a significant secretion of TNF-α from BMDCs and BMDMs (Figure 1D). DSS could also induce caspase-1 activation in BMDCs (Figure 1E), and the incubation of BMDCs with caspase-1 specific inhibitor Z-YVAD-FMK (15 μM) led to a partial, albeit statistically significant decrease in the release of IL-1β (Figure 1F). These results suggest that DSS can induce IL-1β release in murine BMDCs and BMDMs, and that this effect is partially dependent upon caspase-1 activation.

### NLRP12 negatively regulates IL-1β release in NLRP3 inflammasome -independent manner

**Figure 2:**
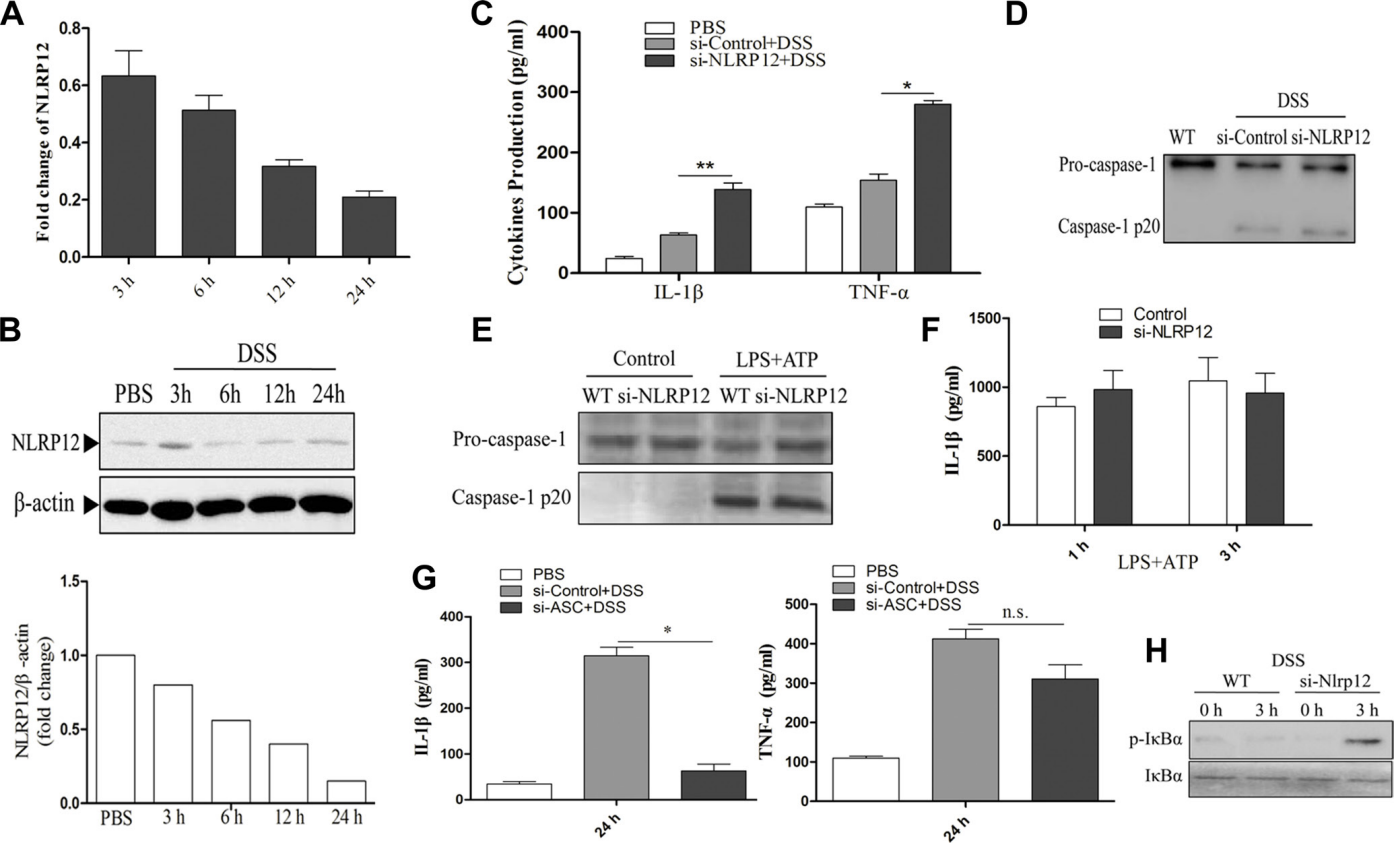
NLRP12 inhibits the release of pro-inflammatory cytokines upon DSS stimulation (**A**) Measurement by quantitative PCR or western blot (**B**) of the expression of NLRP12 in BMDCs at the indicated time points after exposure to DSS. ELISA analysis of IL-1β and TNF-α (**C**) released in cell culture supernatants of NLRP12-knock-down DC2.4 cells upon DSS stimulation. (**D**) Western blot analysis of pro-caspase-1 p45 and bioactive form of caspase-1 p20 in NLRP12-knock-down DC2.4 cells upon DSS treatment. (**E**) Western blot analysis shows the effect of NLRP12 knock-down on the LPS+ATP-induced caspase-1 activation. (**F**) ELISA analysis of the effect of NLRP12 knock-down on LPS+ATP-induced IL-1β release in DC2.4 cells. (**G**) ELISA analysis of IL-1β and TNF-α released in the cell culture supernatants of ASC knock-down DC2.4 cells treated with DSS. (**H**) p-IκBα/IκBα ratio was evaluated in wild-type dendritic cells and NLRP12 knock-down dendritic cells after DSS stimulation. Data were performed in triplicate and expressed as the mean ± SD, and are representative of three separate experiments. *p < 0.05 and **p < 0.01, significantly different from control under the same experimental conditions, n.s., no significant.

It has been reported that NLRP12 could exert both pro- and anti-inflammatory functions, and that NLRP12 deficient mice are more susceptible to DSS induced colon inflammation and tumorigenesis [8, 23]. To further elucidate the mechanism of NLRP12 involvement in the DSS induced colitis, we first examined the expression pattern of NLRP12 upon DSS stimulation in murine dendritic cells. Murine BMDCs were stimulated with DSS at different time points, and the total RNA and protein were extracted to analyze the NLRP12 expression. The results showed that mRNA expression of NLRP12 significantly decreased upon DSS stimulation (Figure 2A). This down-regulatory trend of NLRP12 expression was confirmed at protein level by western blot analysis (Figure 2B). To further illustrate the role of NLRP12 in DSS-induced inflammation, we examined the effect of siRNA-mediated silencing of NLRP12 on the release of pro-inflammatory cytokines. siRNA-mediated disruption of NLRP12 significantly increased the release of IL-1β and TNF-α (Figure 2C) upon DSS stimulation. In addition, knockdown of NLRP12 expression had no significant effect on caspase-1 activation upon DSS stimulation (Figure 2D). These data suggest that the down-regulation of NLRP12 is required for the release of proinflammatory cytokines during DSS induced inflammation.

It has been reported that NLRP3 inflammasome is involved in DSS induced mice colitis [24]. To clarify the effect of NLRP12 down-regulation on NLRP3 inflammasome activation, we examined caspase-1 activation and IL-1β production upon LPS priming and ATP stimulation in NLRP12-knockdown cells. The results indicated that the knockdown of NLRP12 did not affect ATP- induced caspase-1 activation, as there was no significant difference in the cleavage of pro-caspase-1 into active caspase-1 p20 between NLRP12 knockdown and control groups (Figure 2E). Furthermore, NLRP12 knockdown had no significant effect on IL-1β release upon ATP stimulation in dendritic cells (Figure 2F). These results suggest that NLRP12 does not interfere with NLRP3 inflammasome activation in dendritic cells.

As NLRP12 may interact with ASC to form NLRP12 inflammasome which promotes release of pro-inflammatory cytokines [7, 25], we examined the role of ASC in DSS-induced release of pro-inflammatory cytokines in BMDCs. As shown in Figure 2G, ASC down-regulation significantly reduced the release of IL-1β upon DSS stimulation, but had no effect on TNF-α production. On the contrary, NLRP12 down-regulation significantly increased the release of both IL-1β and TNF-α (Figure 2C). This suggests that ASC does not associate with NRLP12 to form the pro-inflammatory NLRP12 inflammasome during DSS stimulation. Interestingly, NLRP12 disruption was shown to increase NF-κB activation as indicated by higher p-IκBα/IκBα ratio in NLRP12-knockdown cells (Figure 2H), which is consistent with a previous report [23]. Collectively, these data suggest that NLRP12 inhibits the release of pro-inflammatory cytokines in DSS-induced cells by interfering with NF-κB activation, and that the inhibitory role of NLRP12 is independent of the assembly of NRLP12 or NLRP3 inflammasome.

### Blimp-1 down-regulates NLRP12 expression during DSS stimulation

**Figure 3:**
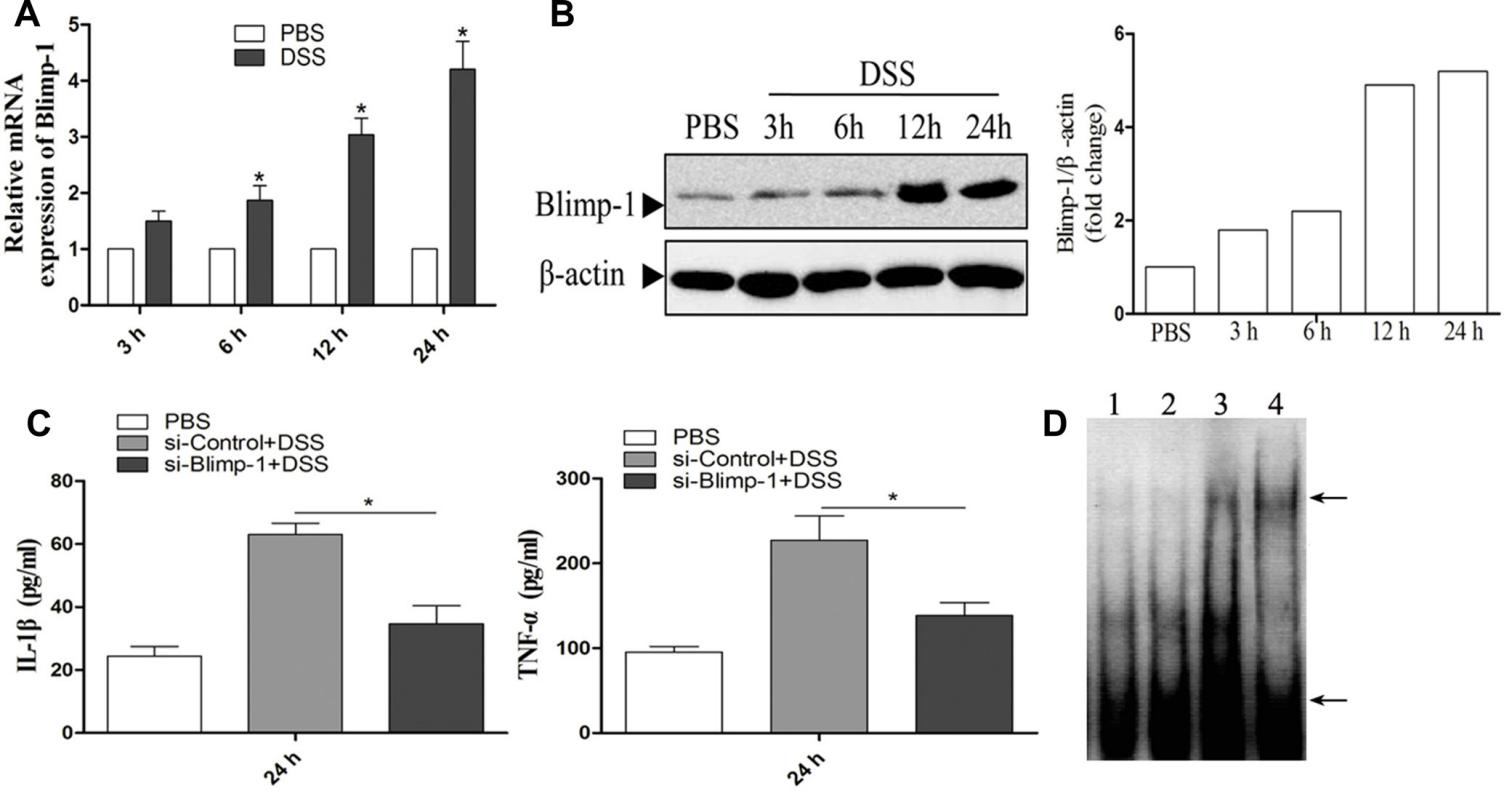
Blimp-1 binds to the NLRP12 promoter and down-regulates its expression during DSS stimulation Measurement by quantitative PCR (**A**) or western blot analysis (**B**) of the expression of Blimp-1 in BMDCs after exposure to DSS. (**C**) ELISA analysis of IL-1β and TNF-α released in cell culture supernatants of Blimp-1-knock-down D2.4 cells treated with DSS. (**D**) EMSA analysis of the Blimp-1 binding site in the NLRP12 promoter (1: free DNA; 2: cells treated with PBS; 3: cells treated with DSS; 4: cells treated with TNF-α for detecting NF-κB as positive control), the top arrow suggest the complex of protein with labeled probe, and the bottom arrow indicate probe alone. Data are mean ± SD of triplicate samples. *p < 0.05, significantly different from control under the same experimental conditions.

It has been reported that Blimp-1 participates in the development of colitis [22], and that Blimp-1 inversely correlates with NLRP12 expression during cell differentiation [26]. We, therefore, set out to investigate the interaction between Blimp-1 and NLRP12 in DSS-induced mice colitis. We first examined the effect of DSS treatment on the mRNA expression of Blimp-1 in BMDCs, and found that DSS treatment significantly up-regulated the mRNA expression of Blimp-1 at the examined time points (Figure 3A). This expression pattern of Blimp-1 was confirmed at protein level by western blot analysis (Figure 3B).

Next, we analyzed the effect of siRNA-mediated silencing of Blimp-1 on the release of pro-inflammatory cytokines in DSS treated DCs. siRNA treatment significantly down-regulated Blimp-1 expression at protein levels (Supplementary Figure S1). Following Blimp-1 silencing, DC2.4 cells were treated with DSS for 24 hours, and the cell-culture supernatants were collected and used to examine the release of pro-inflammatory cytokines by ELISA. The siRNA-mediated silencing of Blimp-1 significantly reduced the release of IL-1β and TNF-α after exposure to DSS (Figure 3C), suggesting a key role of Blimp-1 in promoting DSS-induced pro-inflammatory response.

To examine whether NLRP12 and Blimp-1 directly interact after exposure to DSS, nuclear extracts were prepared from BMDCs stimulated with DSS for 24 h and analyzed by EMSA. The EMSA result showed a significantly higher DNA binding activity in DSS stimulated cells compared to control group (Figure 3D). Collectively, these data indicate that Blimp-1 can bind to NLRP12 promoter and down-regulate its expression during DSS stimulation.

### TLR4 is involved in Blimp-1 mediated NLRP12 down-regulation in DSS-induced mice colitis

**Figure 4:**
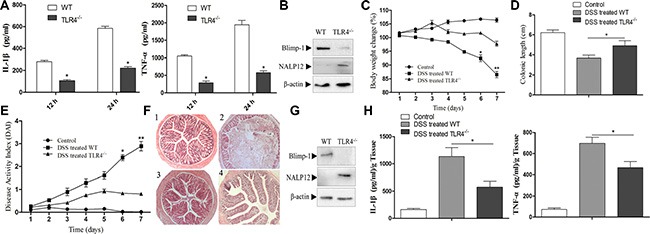
TLR4 participates in Blimp-1 mediated NLRP12 down-regulation in DSS-induced mice colitis (**A**) ELISA analysis of IL-1β and TNF-α secretion by BMDCs from wild-type and TLR4^−/−^ mice in response to DSS treatment. (**B**) Western blot analysis of Blimp-1 and NLRP12 expression in BMDCs from wild-type and TLR4^−/−^ mice upon DSS stimulation. (**C–E**) Body weight (C) and Diseases Activity Index (E) were scored daily, and colonic length (D) was measured at the seventh day of DSS administration. (**F**) Representative images of H&E staining of colon section from (1) PBS control, (2) wild-type treated with DSS, (3) TLR4^−/−^ mice, and (4) TLR4^−/−^ at the seventh day of DSS treatment. (**G**) Blimp-1 and NLRP12 levels were evaluated in colons harvested from wild-type and TLR4−/− mice at day 7 after colitis initiation. (**H**) Distal colons collected at the seventh day of DSS administration were used to determine the level of pro-inflammatory cytokines IL-1β and TNF-α. Data were as the mean ± SD, and are representative of three separate experiments. *p < 0.05 and **p < 0.01, significantly different from control under the same experimental conditions, *n* = 6.

Several studies have shown that TLR4 is implicated in DSS-induced mice ulcerative colitis and colitis-associated colorectal tumors. Blocking TLR4 can prevent the progress of DSS-induced colitis, and TLR4 deficient mice were markedly protected from colon carcinogenesis [27, 28]. However, there has been no report addressing the relationship between TLR4 and Blimp-1 or NLRP12 during the development of colitis. To address this question, we first examined the role of TLR4 in DSS-stimulated pro-inflammatory response. TLR4 deficient BMDCs and wild type control were stimulated with DSS for 12 h or 24 h. TLR4 deletion significantly decreased IL-1β and TNF-α release upon DSS treatment (Figure 4A). We then examined the expression of Blimp-1 and NLPR12 in TLR4 deficient cells after DSS treatment. The results showed that TLR4 deletion significantly decreased Blimp-1 and increased NLRP12 expression upon DSS stimulation (Figure 4B), suggesting that TLR4 may act synergically with Blimp-1 to inhibit NLRP12 expression and promote proinflammatory response during DSS stimulation.

Unsurprisingly, DSS-treated group had significantly greater weight loss compared to the control group from 5 to 7 days, whereas mice deficient in TLR4 showed a less pronounced weight loss (Figure 4C). As a marker of colonic inflammation, colon length was measured by day 7; the colon length of the DSS group was shorter compared to the control group and the TLR4 deficient group (Figure 4D). The results of the daily measured Disease Activity Indices (DAI), comprising weight loss, occult blood and stool consistency, were also consistent with the *in-vitro* results (Figure 4E). Finally, histological examination also demonstrated that TLR4 deficiency significantly decreased DSS-induced colonic ulceration and inflammation (Figure 4F). These data provide additional evidence of the role of TLR4 in promoting the pro-inflammatory response that is associated with DSS-induced mice colitis.

To further clarify the relevance of TLR4 in the regulation of Blimp-1 and NLRP12 expression *in vivo*, we examined the expression of Blimp-1 and NLRP12 in colon tissues collected from TLR4-deficient and wild type mice exposed to DSS treatment. As shown in Figure 4G, TLR4 deficient mice had lower Blimp-1 expression and higher NLRP12 expression compared to wild type group after 7-day DSS administration. Finally, we evaluated the level of IL-1β and TNF-α in colon tissues, and found a significant decrease of IL-1β and TNF-α in TLR4 deficient mice compared to control 7 days after the initiation of colitis (Figure 4H). These findings are consistent with *in vitro* results and further confirm the involvement of TLR4 in the regulation of the expression of Blimp-1 and NLRP12 during mice colitis.

## DISCUSSION

**Figure 5:**
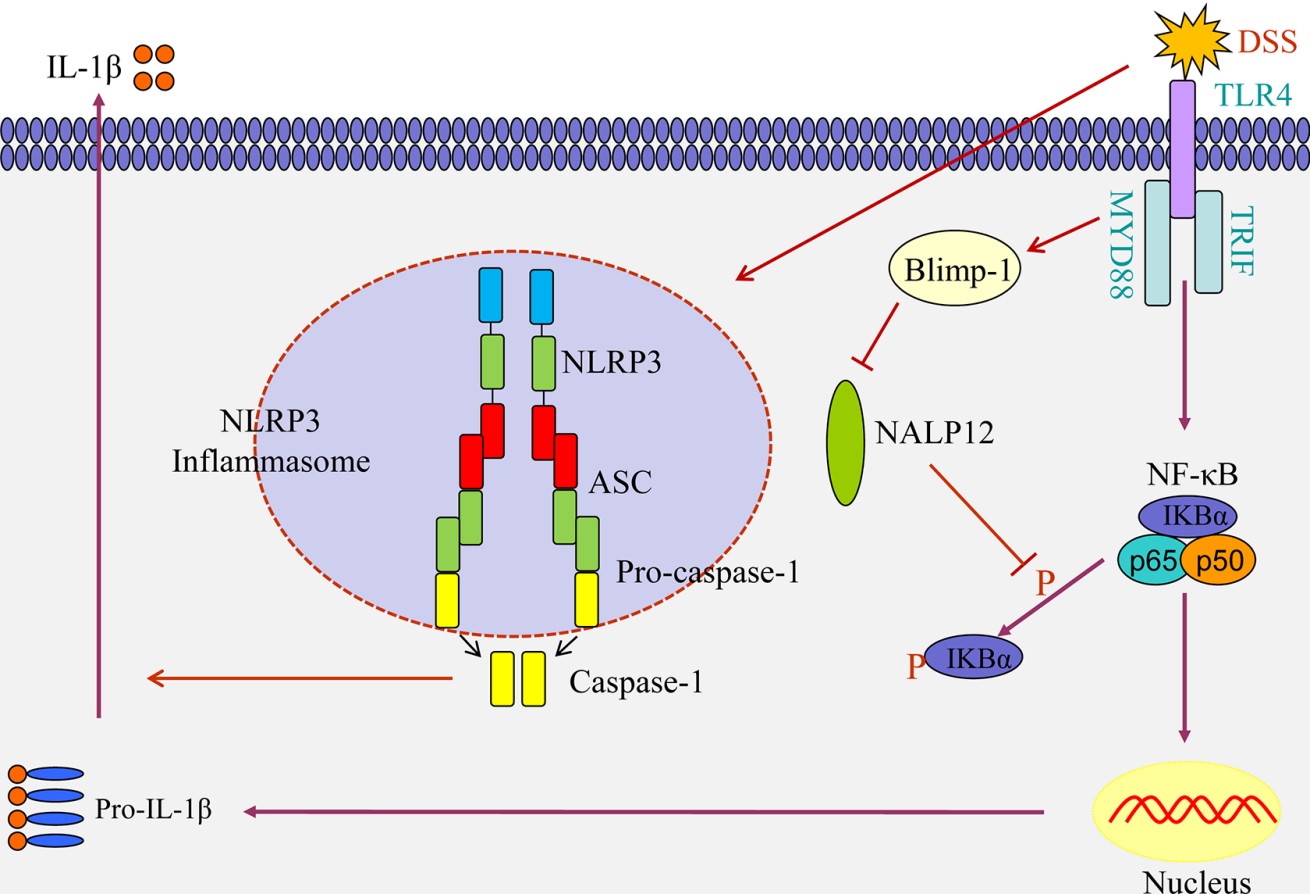
Schematic diagram illustrating the hypothetical molecular signaling triggered by DSS stimulation DSS leads to NLRP12 decrease through up-regulation of Blimp-1 in dendritic cells. Down-regulation of NLRP12 increases NF-κB activation, which results in an increase in Pro- IL-1β level. Meanwhile, DSS-induced NLRP3 inflammasome activation leads to caspase-1-mediated maturation of IL-1β.

Mice model of ulcerative colitis have been used to investigate the regulatory mechanisms involved in the disease development. Previous studies have shown that NLRP12 may act as a negative regulator during DSS-induced mice colitis [8, 23]. However, the upstream factors involved in regulation of NLRP12 expression and function during colitis have not yet been identified. In this study, we found (i) that NLRP12 inhibits IL-1β release during DSS-induced mice colitis in NLRP3 inflammasome-independent manner; (ii) that Blimp-1 down-regulates NLRP12 expression during DSS stimulation; and (iii) that TLR4 participates in Blimp-1-mediated NLRP12 down-regulation during the development of mice colitis. Based on these findings, a schematic diagram of the hypothetical molecular signaling triggered by DSS stimulation is drawn in Figure 5.

NLRP12 has been shown to play an important role in DSS-induced ulcerative colitis. NLRP12 was first reported to promote release of pro-inflammatory cytokines by forming NLRP12 inflammasome with ASC and pro-caspase-1 [7, 25]. On the other hand, NLRP12 could also exert inhibitory effect during the development of colitis. Zaki et al. and Allen et al. reported that NLRP12 suppresses colon inflammation and tumorigenesis through negative regulation of the downstream factors NF-κB and ERK signaling [8, 23], which is consistent with our results according to which down-regulation of NLRP12 is a prerequisite for pro-inflammatory cytokines release during DSS stimulation in mice BMDCs. NLRP12 anti-inflammatory function during DSS-induced mice colitis seems to be not related to NLRP12 inflammasome assembly. However, it would be of great interest to investigate the factors that may influence the balance between NLRP12 anti-inflammatory effect and the inflammasome associated pro-inflammatory effect during the development of colitis.

Blimp-1 has long been identified as a “master regulator” of cell differentiation [29]. Christopher and co-workers reported that increased Blimp-1 expression leads to the reduction of NLRP12 expression during PMA-mediated differentiation [26]. In our study, we showed that Blimp-1 is required for DSS-induced pro-inflammatory response, and that it exerts its pro-inflammatory role by directly binding to NLRP12 promoter to down-regulate its expression. These results are in sharp contrast with those reported by a recent study according to which mice deficient in Blimp-1 had more effector CD4+ and CD8+ in the periphery and developed severe colitis, suggesting a context dependent role of Blimp-1 in the regulation of inflammatory response during the development ulcerative colitis. Further studies are surely needed before any conclusion regarding the net effect of Blimp-1-mediated regulatory role on inflammatory response during the development of colitis may be drawn.

TLR4 is known to play an important role in the development of colitis, and was shown to promote colon carcinogenesis in chronic colitis. Blocking TLR4 by a specific antibody can prevent the development of DSS-induced colitis by targeting the TLR4-P38MAPK-c-jun pathway [27]. TLR4 agonists, such as LPS, were shown to strongly up-regulate Blimp-1 mRNA expression in murine splenic B cells and B-1 cells from the peritoneal cavity [30]. Our results further illustrated the critical role of TLR4 and Blimp-1 interaction in the development of colitis. TLR4 knockout dendritic cells failed to up-regulate Blimp-1 expression, which cleared the way for the up-regulation of NLRP12 expression and decreased the inflammatory responses upon DSS stimulation.

In conclusion, we have identified the previously unrecognized regulatory axis of TLR4-Blimp-1-NLRP12 that modulates inflammatory response in dendritic cells during DSS-induced mice colitis. Although more studies are needed *in vivo* and *in vitro* to confirm these initial findings, by identifying the upstream regulators of NLRP12 in DSS-induced colitis, our observations enhance our understanding of the mechanisms lying behind colitis development, and identified potential molecular targets for the development anti-inflammatory therapy of colitis through the targeting of the TLR4-Blimp-1-NLRP12 axis.

## MATERIALS AND METHODS

### Reagents

Rabbit anti-mouse NLRP12, Blimp-1, IκBα and p-IκBα antibody were acquired from Santa Cruz Biotechnology (Cambridge, MA, USA); Rabbit anti-mouse caspase-1 antibody were from BioVision (CA, USA) and mouse anti-mouse β-actin antibody were from Abbkine (Redlands, CA, USA). Lipopolysaccharide (LPS) and ATP were purchased from Sigma-Aldrich (St. Louis, MO, USA). The caspase-1 specific inhibitor Z-YVAD-FMK was from BioVision (CA, USA). ELISA kits for mouse interleukin 1β and TNF-α were from MultiSciences (Hangzhou, China) and Fast Protein Precipitation and Concentration Kit were from Aidlab Biotechnology (Beijing, China). Electrophoretic Mobility Shift Assay Kit was from Viagene Biotech (Changzhou, China). Reagents and apparatus used in western blot were obtained from Bio-Rad (Hercules, CA, USA); The Goat anti-rabbit or mouse secondary antibody were from Abbkine (Redlands, CA, USA).

### Cell culture and treatments

Bone marrow was isolated from WT and TLR4^−/−^ mice as described previously [31]. BMDM, BMDC and immortalized cell line DC2.4 (originating from C57BL/6 mice) were cultured in a humidified incubator at 37°C with 5% CO2 in DMEM/F12 medium (Hyclone, Logan, UT, USA) supplemented with 10% fetal bovine serum (Gibco, NY, USA), 100 μg/ml streptomycin and 100 U/ml penicillin (Gibco).

### Enzyme-linked immunosorbent assay for IL-1β and TNF-α secretion

Tissue was homogenized at low temperature kept by liquid nitrogen and centrifuged at 900 × g at 4°C for 10 min. Supernatants were collected for the detection of the release of proinflammatory cytokines IL-1β and TNF-α. Cell-culture supernatants were collected at indicated time points. The levels of cytokines in supernatants were assayed by ELISA using commercial kits (MultiSciences Biotech) in accordance with the manufacturer's instructions.

### Animal model

6- to 8- week old male C57BL/6 mice and TLR4 deficient mice were obtained from the Laboratory Animal Center of Zhejiang University (Hangzhou, China) and Model Animal Research Center of Nanjing University (Nanjing, China), respectively. Animal experiments were approved by the Institutional Animal Care and Use Committee of Zhejiang University. Mice were acclimatized at least 7 days before experiments. Acute colitis was induced with 5% DSS (molecular weight of 40,000 Da; MP Biomedicals, Solon, OH) dissolved in drinking water given ad libitum for 7 day (*n* = 6/group) as described in literature [32]. Control mice were given tap water. Colonic tissues were collected, and homogenized in RIPA buffer and subjected to western blot and ELISA. Some colonic tissues were used for H&E staining to detect colonic lesions [33].

### Disease activity index

The disease activity index was assessed according to a standard scoring system [34]. The clinical score was assessed by an investigator blinded to the protocol. Body weight, presence of occult blood, and stool consistency were examined daily. Briefly, no body weight loss was scored as 0, weight loss of 1–5% 1 point, 6–10% 2 points, 11–20% 3 points, and more than 20% 4 points. For occult blood, no blood was assigned 0, 2 points for positive and 4 points for gross bleeding. For stool consistency, 0 points were assigned for well-formed pellets, 2 points for pasty stools that did not adhere to the anus, and 4 points for liquid stools that adhere to the anus [35].

### Histopathology

The distal colons were fixed in 10% buffered formalin for histological analysis. Samples were embedded in paraffin, sectioned at 5 μm, and processed for H&E staining.

### Electrophoretic mobility shift assay (EMSA)

BMDC-derived nuclear proteins were used for EMSA according to previously described data [36]. Biotin-labeled double-stranded oligonucleotides containing the NLRP12 binding site were used as a probe (forward: 5′- ACAGCAGAAGTGAAAATCTTTTTCA-3′, reverse: 5′- TGAAAAAGATTTTCACTTCTGCTGT-3′). TNF-α stimulated cells examined for detecting NF-κB were used as positive control (forward: 5′- AGTTGAGGGGA CTTTCCCAGGC -3′, reverse: 5′- GCCTGGGAAAGT CCCCTCAACT-3′). EMSA were carried out using the LightShift Chemiluminescent EMSA Kit (Thermo Scientific) in accordance with the manufactuer's instructions. Each binding reaction contained 4 μg nuclear protein lysates, 60 ng poly (dI–dC), 1 × binding buffer and 50 fmol biotin-labeled oligonucleotide in a final total volume of 20 μl. After 20 min incubation at room temperature, the binding reactions were subjected to non-denaturing PAGE on 6% DNA retardation gels (Life Technologies), and then transferred to nylon membranes. The biotin labeled DNA-proteins were visualized by chemiluminescene.

### SiRNA transfections and treatments

SiRNA used for NLRP12, Blimp-1 and ASC silencing, and the scramble siRNA sequence used as control were purchased from Qiagen (Valencia, CA). For siRNA transfections, DCs were plated at 6 × 10^4^ cells/well in a 12-well plate, and transfected the next day in accordance with the manufacturer's instructions. Briefly, 100 ng siRNA (NLRP12, Blimp-1, ASC and control, respectively) were diluted in 100 μl culture medium without serum on the day of transfections. A volume of 3 μl of Hiperfect Transfection Reagent (Qiagen) was added to the diluted siRNA and mixed by vortex. The mixture was added onto the cells after 5 to 10 minutes at room temperature. The efficiency of siRNA-mediated disruption was evaluated by quantitative PCR and western blot analysis, respectively.

### Quantitative PCR

Total RNA was extracted using Total RNA Isolation Kit (Aidlab Biotechnology), and reverse transcribed into cDNA using cDNA Synthesis Kit (Fermentas, MD, USA) in accordance with the manufacturer's instructions. Quantitative PCR (qPCR) was performed using SYBR Green Master mix (Biorad, Hercules, USA) in a thermal cycler ABI 7300 (Applied Biosysterms, CA, USA) with the following primers: NRLP12, 5′- GGGTCACTCCAAATAATGGT-3′ and 5′- GGAATTTCCTTCGGACATAG-3′; Blimp-1, 5′-GAG TACATACCGAAGGGAACA-3′ and 5′- CATCAATGAA GTGGTGGAAC-3′; β-actin, 5′- CCTTCTGACCCATTCC CACC-3′ and 5′-GCTTCTTTGCAGCTCCTTCG -3′. Quantification was determined using the comparative CT method (2^−ΔΔCT^) [37]. All samples were analyzed in triplicate.

### Western blot analysis

Cytoplasmic proteins from colonic tissues and cells were extracted using a protein extract kit (Aidlab Biotechnology). Equal amounts of protein (50 μg in each lane) were electrophoresed in SDS-PAGE on 12% gels. The separated proteins were transferred onto PVDF membrane (Millipore Corp., Bedford, MA). The membrane was incubated overnight at 4°C with antibodies for NLRP12, Blimp-1, IκBα, p-IκBα, caspase-1 and β-actin. Then the membranes were incubated with secondary antibodies. Bands of immune-reactive protein were detected by an image system (Clinx Science Instruments, China).

### Statistical analysis

All assays were performed on three separate occasions. Results are expressed as means ± SD. All comparisons of data were made using one-way ANOVA followed by Student's *t*-test or *post-hoc* Turkey's test. Prism GraphPad 5.0 (San Diego, CA, USA) was used, and *P* values of < 0.05 were considered significant.

## SUPPLEMENTARY MATERIALS FIGURE


