# Supplementary material for: Inflammasome-independent role of NLRP12 in suppressing colonic inflammation regulated by Blimp-1
Source: Oncotarget. 2016 Apr 20;7(21):30575–84. doi: 10.18632/oncotarget.8872 (PMC5058702; doi:10.18632/oncotarget.8872)
Supplement: Supplementary file 1 [file oncotarget-07-30575-s001.pdf]

Inflammasome-independent role of NLRP12 in suppressing colonic inflammation regulated by Blimp-1

Supplementary Materials

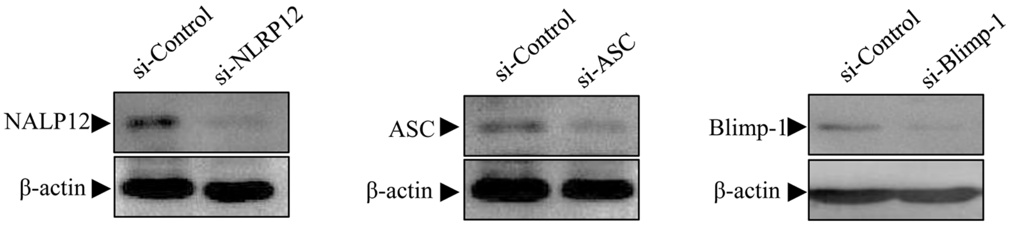

Supplementary Figure S1: Western blot analysis of the protein expression of NLRP12 (A), ASC (B) and Blimp-1 (C) in DC2.4 cells after siRNA transfection. Cells were transfected with specific siRNA (si-NLRP12, si-ASC and si-Blimp-1), or with scramble control siRNA (si-Control). Proteins were evaluated by western blot analysis.
